# Adaptation to Abundant Low Quality Food Improves the Ability to Compete for Limited Rich Food in *Drosophila melanogaster*

**DOI:** 10.1371/journal.pone.0030650

**Published:** 2012-01-24

**Authors:** Roshan K. Vijendravarma, Sunitha Narasimha, Tadeusz J. Kawecki

**Affiliations:** Department of Ecology and Evolution, University of Lausanne, Switzerland; Vetmeduni Vienna Institute of Population Genetics, Austria

## Abstract

The rate of food consumption is a major factor affecting success in scramble competition for a limited amount of easy-to-find food. Accordingly, several studies report positive genetic correlations between larval competitive ability and feeding rate in *Drosophila*; both become enhanced in populations evolving under larval crowding. Here, we report the experimental evolution of enhanced competitive ability in populations of *D. melanogaster* previously maintained for 84 generations at low density on an extremely poor larval food. In contrast to previous studies, greater competitive ability was not associated with the evolution of higher feeding rate; if anything, the correlation between the two traits across lines tended to be negative. Thus, enhanced competitive ability may be favored by nutritional stress even when competition is not intense, and competitive ability may be decoupled from the rate of food consumption.

## Introduction

Food is often a limiting factor for animals and an object of intense competition. In particular, competition for a limited amount of high quality food is likely to favour monopolization of resources through territoriality or social dominance as well as faster feeding and development. However, malnutrition may also result from food being of poor quality even if available in large amount. This type of nutritional stress may favour different adaptations than competition for high-quality food, such as efficient food processing and utilization.

Adaptation to both types of nutritional stress has been addressed with experimental evolution in *Drosophila*, where larvae in nature develop on ephemeral food patches of varying quality and often compete with other larvae [1], [2]. Experimental populations of *D. melanogaster* maintained under high larval density on high-quality food have evolved accelerated development, faster growth and increased competitive ability for food [3], [4]. This increased competitive ability, quantified as relative survival to adulthood of two strains competing for a small amount of food, appears to trade-off with lower energetic efficiency. This trade-off raises the question of whether greater competitive ability would also be favoured under nutritional stress resulting from low food quality rather than quantity, where the efficiency of food use might be more important than the scramble competition.

Here we address this question using six *D. melanogaster* populations adapted to chronic larval malnutrition as a result of being maintained for 84 generations under low density on an extremely poor larval food [5]. Similar to populations adapted to larval crowding, these populations have evolved faster development [5]. However, faster development does not automatically lead to competitive advantage [6]. Furthermore, in contrast to the crowding-adapted populations [3], [4], these populations do not show faster growth than unselected controls under good food conditions [7]. The question of their competitive ability thus remains open. Larval competitive ability in *Drosophila* is thought to be mediated to a significant degree by a higher feeding rate [8], [9]. Consistent with this, the experimental evolution of higher competitive ability in crowded populations has been coupled with an increase in larval feeding rate [4], [9], [10]. Conversely, larvae from populations selected for faster development [11], [12], [13] and parasitoid resistance [14], [15] have evolved both lower larval competitive ability and lower rates of larval feeding. Finally, fast-feeding *D.melanogaster* larvae from a bi-directional selection experiment on larval feeding rate were better competitors than both slow-feeding larvae and unselected controls [16]. Under scramble competition for high-quality food, a high feeding rate allows the individual to obtain more food before it is consumed by competitors. However, a higher rate of food intake might also be favoured in the absence of competition as a way to compensate for low nutritional content of food. Therefore, we also test if larvae from the selected populations show a higher rate of food intake than controls, and if variation in food intake and competitive ability correlates across replicate populations within selection regimes.

## Materials and Methods

### Experimental evolution

Six populations of *D.melanogaster* (referred to as selected populations) were reared on poor larval food for 84 generations with time-to-emergence restricted to 14 days; six control populations were reared on standard food [5]. Both regimes were maintained at 25°C, 70% humidity and at a density of 200 eggs/30 ml food (also the conditions used in this study). The poor food contained ¼ of the amounts of sugars, yeast and cornmeal of the standard food. The selected populations adapted to this chronic larval malnutrition by evolving increased egg-to-adult viability, smaller critical size for metamorphosis initiation, smaller adult body size and faster development [5], [7], [17]. Before the assays reported here, all populations were reared on standard food for two generations before the assays to remove effects of maternal environment.

### Larval competitive ability

Larval competitive ability of selected and control *D. melanogaster* populations was determined by competing them against a “ester”genotype (brown-eyed *sepia* mutant), following the protocol established in previous studies [15], [18], [19]. The assay was done in 120 vials with 10 ml agar (2%) layered with 0.2 ml of 25% live yeast suspension [19]. Eggs from *sepia* flies were collected over 3 h and 20 eggs per vial were set up and incubated. Twenty-four hours later eggs were collected from the six selected and six control populations over a 3 h period. Groups of 10 eggs per population was added to 10 replicate vials already containing 20 *sepia* larvae and incubated for 18 days. The number of wild-type (i.e., selected or control) flies (*x*) and the number of *sepia* (tester) flies (*t*) that survived to adulthood in each vial were scored. The competitive index (CI) was calculated for each vial as log((2*x*+1)/(*t*+1)) (modified from [18]). A competitive index of zero indicates equal survival of experimental and tester larvae.

### Larval feeding rate

Traditionally, cephalopharyngeal retractions were used to quantify feeding rate [20], [21], but the absence of correlation between mouth-hook movement and amount of food ingested [22] questions the reliability of this method. We thus use a newer dye-based method [22] instead. Three bottles containing standard food and 200 eggs (collected over a 3 h period) were set up for each of the 12 populations. After 92 h of incubation, groups of 50 larvae were collected per bottle and allowed to feed on 50% yeast paste coloured with 0.16% Erioglaucine dye (FD&C Blue No. 1, Sigma) for 15 minutes, in Petri-plates lined with agar. A fourth group of 50 larvae per population was collected from one of the three bottles at random, allowed to feed on uncolored yeast paste for 15 minutes and used to measure background OD. Each group of larvae was then washed twice in distilled water, placed in 1.5 ml eppendorf tubes with a few glass beads, and flash-frozen in liquid nitrogen. Larvae within each tube were homogenized in 250 µl distilled water and centrifuged at 13 g for 10 min; 225 µl of supernatant was transferred to a new 1.5 ml tube containing 50 µl 100% ethanol. Tubes were then vortexed for 30·seconds and re-centrifuged for 10·min. 225 µl of supernatant was centrifuged at 13 g for 5 min in a new tube and 150 µl of this supernatant was loaded into a 96-well crystal plate. The relative amount of dye ingested by each group was quantified as the optical density (OD) of the sample at 633 nm (Spectramax 190) with background OD of respective population subtracted. Additionally, from each bottle 10 more larvae were collected, washed, dried on paper towels, and weighed as a group to the nearest microgram on a Mettler MT5 balance to exclude differences in larval body size as a confounding factor.

### Statistical analysis

The values of competitive index, feeding rate and larval weight for each replicate were analyzed with a mixed model ANOVA using JMP 8.0, where regime (selected or control) was a fixed factor and replicate population was a random factor nested within the regime. We also tested separately for a difference between the selected and control lines in survival in the competitive assay, and for the survival of the *sepia* tester flies when competing with selected versus control flies. For this we used a generalized linear model with lines nested within regimes, binomial error distribution and a logit link function.

An analysis of covariance using mean trait values for each population was performed to test if the feeding rate among populations co-varies with competitive ability. Competition index was the response variable, regime was the experimental factor, and feeding rate was the covariate.

## Results

**Figure 1:**
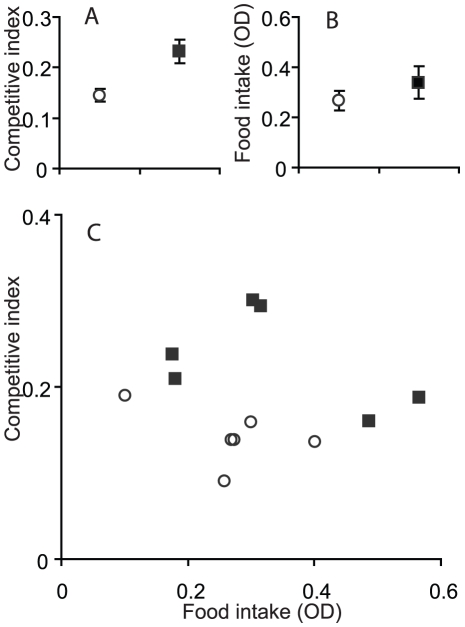
Larval competitive ability and feeding rate of the selected (squares) and control (circles) populations. (A) Mean competitive index and (B) mean food intake for each regime (bars indicate ± one standard error based on variation among populations). (C) Correlation plot of estimates of the two variables for each replicate population.

Despite the “tester” competitor *sepia* having a head-start, larvae from both regimes survived better than the “testers”, as indicated by positive values of competitive index. The competitive index was greater for larvae from selected than control populations (F_1,10_ = 11.2, P = 0.007; Fig. 1A), while variation among the replicate populations was not significant (F_10,108_ = 0.9, P = 0.54). However, the proportion of individuals surviving to adulthood did not differ between the regimes (selected 0.525±0.028, control 0.538±0.032; χ^2^_1_ = 0.2, *P* = 0.64). Rather, the difference in competitive index was largely due to the lower survival of the tester *sepia* larvae when competing with the selected versus control lines (0.306±0.021 versus 0.362±0.020; χ^2^_1_ = 9.3, *P* = 0.0022).

The amount of coloured yeast ingested by the larvae in 15 minutes, measured as OD was not different between the selection regimes (*F*_1,10_ = 0.3, *P* = 0.6), but varied among replicate populations (*F*_10,23_ = 4.7, *P* = 0.001) (Fig. 1B). There was a trend within both regimes for populations with higher feeding rates to have lower competitive ability (Fig. 1C). While suggesting a negative relationship between larval competitive ability and feeding rate (slope −0.17), this trend was not significant (*F*_1,9_ = 2.9, *P* = 0.13); the significant difference in competitive index between selection regimes was confirmed (*F*_1,9_ = 13.2, *P* = 0.005).

We found no differences in the wet weight of larvae at the time of feeding rate assays between selected (0.62±0.01 µg) and control populations (0.64±0.17 µg; *F*_1,10_ = 0.8, *P* = 0.39), or among replicate populations (*F*_10,24_ = 1.8, *P* = 0.12).

## Discussion

Over 84 generations of selection the study populations have been evolving under low-quality but relatively abundant food; under the selection regime the total energy content of food available per larva was about 10 times the energy content of a pre-pupation larva [23]. Yet, this study shows that these selected populations have evolved a stronger ability to compete for a very limited amount of high-quality food, which represents a very different type of nutritional stress. Enhanced competitive ability in *Drosophila* has so far only been reported from populations evolved under crowded conditions [3], [4]. In contrast, several other experimental selection regimes – for fast development [6], resistance to pathogens [19] and parasitoids [14], [15], or improved associative learning [24] – have led to a decrease in the competitive ability. Thus, our results are rather unexpected and suggest that the previously reported trade-off between competitive ability and energetic efficiency [4] has been of little importance in our selected populations.

It remains an open question whether the improved competitive ability was directly under selection under the poor food regime, despite the low density, or whether it is a by-product of evolutionary changes in other traits. The selected populations have evolved a smaller critical size for pupation initiation and complete pupation at substantially smaller size [7], and so they would presumably need less energy and nutrients to survive to adulthood. However, this does not explain their higher competitive index – they do not survive better in the competitive assays than the control populations. Rather, larvae from the selected populations exert stronger negative effects than controls on survival of the tester genotype. A potential explanation of this result would have been a higher rate of food consumption by larvae from the selected versus control populations, leaving less food for the tester larvae. However, the selected populations did not show consistently higher rates of food intake than controls, and the correlation among populations between food intake rate and competitive index tended to be negative. This is another unexpected result – previous studies found a close association between feeding rate and competitive ability (see Introduction).

We can thus only speculate about the mechanism of the stronger competitive impact of the larvae from the selected lines on the testers. The number of larvae surviving to adulthood did not differ between the selected and control populations. Nonetheless, among the larvae that did not survive, those from selected populations might have died later than those from the control populations. If so, the *sepia* larvae competing with larvae from a selected population would have transiently faced a larger number of competitors. However, other factors such as differences in foraging behaviour (e.g., the ability to find the best remaining microsites in the food paste), the time spent not feeding (e.g., wandering or moulting), toxicity or waste products or direct antagonistic interactions might have also contributed to this result.

Irrespective of the underlying mechanism, this study shows that a greater ability to compete for a limited amount of high-quality food may be favoured under chronic malnutrition at a rather low population density. Furthermore, it indicates that a higher competitive ability in *Drosophila* larvae can be decoupled from the rate of food intake. Finally, these results suggest that adaptive evolutionary change under some experimental regimes may be most readily apparent by examining effects on the fitness of competitors rather than that of the focal individuals.
